# Macrophage polarization and metabolism in atherosclerosis

**DOI:** 10.1038/s41419-023-06206-z

**Published:** 2023-10-20

**Authors:** Pengbo Hou, Jiankai Fang, Zhanhong Liu, Yufang Shi, Massimiliano Agostini, Francesca Bernassola, Pierluigi Bove, Eleonora Candi, Valentina Rovella, Giuseppe Sica, Qiang Sun, Ying Wang, Manuel Scimeca, Massimo Federici, Alessandro Mauriello, Gerry Melino

**Affiliations:** 1 Department of Experimental Medicine, TOR, University of Rome Tor Vergata, Rome, Italy; 2 The First Affiliated Hospital of Soochow University, Institutes for Translational Medicine, State Key Laboratory of Radiation Medicine and Protection, Suzhou Medical College of Soochow University, Suzhou, China; 3 Department of System Medicine, University of Rome Tor Vergata, Rome, Italy

**Keywords:** Atherosclerosis, Translational research

## Abstract

Atherosclerosis is a chronic inflammatory disease characterized by the accumulation of fatty deposits in the inner walls of vessels. These plaques restrict blood flow and lead to complications such as heart attack or stroke. The development of atherosclerosis is influenced by a variety of factors, including age, genetics, lifestyle, and underlying health conditions such as high blood pressure or diabetes. Atherosclerotic plaques in stable form are characterized by slow growth, which leads to luminal stenosis, with low embolic potential or in unstable form, which contributes to high risk for thrombotic and embolic complications with rapid clinical onset. In this complex scenario of atherosclerosis, macrophages participate in the whole process, including the initiation, growth and eventually rupture and wound healing stages of artery plaque formation. Macrophages in plaques exhibit high heterogeneity and plasticity, which affect the evolving plaque microenvironment, e.g., leading to excessive lipid accumulation, cytokine hyperactivation, hypoxia, apoptosis and necroptosis. The metabolic and functional transitions of plaque macrophages in response to plaque microenvironmental factors not only influence ongoing and imminent inflammatory responses within the lesions but also directly dictate atherosclerotic progression or regression. In this review, we discuss the origin of macrophages within plaques, their phenotypic diversity, metabolic shifts, and fate and the roles they play in the dynamic progression of atherosclerosis. It also describes how macrophages interact with other plaque cells, particularly T cells. Ultimately, targeting pathways involved in macrophage polarization may lead to innovative and promising approaches for precision medicine. Further insights into the landscape and biological features of macrophages within atherosclerotic plaques may offer valuable information for optimizing future clinical treatment for atherosclerosis by targeting macrophages.

## Facts

- Macrophages metabolic characteristics and polarization play a crucial role in the initiation, growth, rupture, and healing stages of atheromatic plaque formation.
- Hypoxia-related changes in macrophage metabolism and phenotype modulate atheromatic plaque growth by HIF-1α expression.
- β-cyclodextrin treatment favor plaque regression promoting cholesterol efflux and anti-inflammatory properties by modulating macrophages metabolism.
- The efferocytosis of apoptotic macrophages lead to immune suppression or inflammation resolution in atheromatic plaques.

## Open Questions

- What are the specific metabolic characteristics of macrophages that contribute to reduce plaque progression and stabilize vulnerable lesions in humans?
- How do macrophage metabolic shifts in response to plaque microenvironmental factors affect the progression or regression of atherosclerosis?
- Can targeting macrophage polarization pathways lead to effective and selective treatments for atherosclerosis?
- How the identification of novel therapeutic targets to regulate the metabolism and polarization of macrophages at plaque sites may lead to innovative therapies for cardiovascular disease leading to effective precision medicine ?

## Introduction

**Fig. 1:**
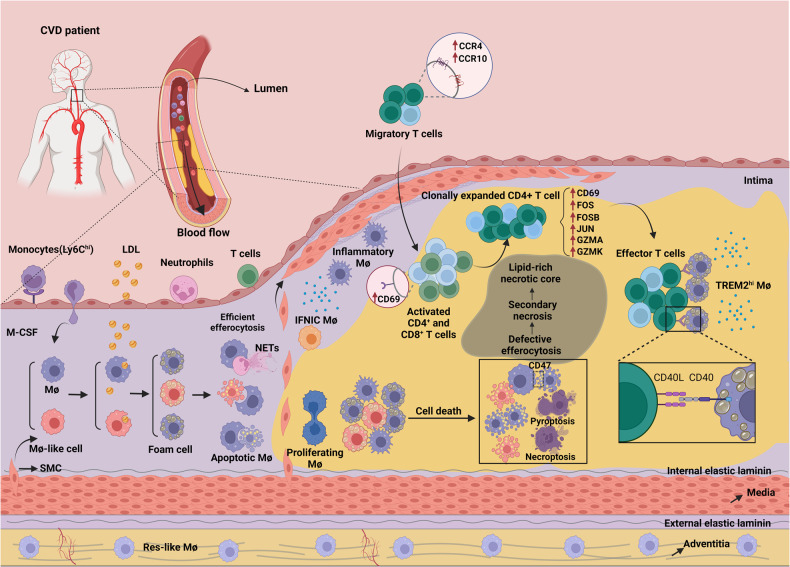
The pathological mechanisms underlying atherosclerosis with autoimmune-like features. A normal artery consists of three layers, the tunica adventitia, tunica media (containing abundant SMCs), and tunica intima (located in the subendothelial space). Under homeostatic conditions, almost no blood leukocytes accumulate in the endothelial layer. In contrast, when activated by proinflammatory cytokines or other cardiovascular risk factors, endothelial cells express leukocyte adhesion molecules (such as ICAM-1 and VCAM-1) and thereby cause the rolling and attachment of circulating monocytes, neutrophils and lymphocytes. In parallel, increased endothelium permeability promotes the entry of lipoproteins into the intima. Monocytes are recruited to the intima by chemokines (such as CCL2) and differentiate into mature macrophages mediated by M-CSF. Within the intima, macrophages take up excessive lipoproteins and form foam cells, which is a crucial step in initiating atherosclerosis. Recent evidence has demonstrated that foam cells can also be formed from macrophage-like cells that are produced via the transdifferentiation of SMCs. Furthermore, excessive lipid metabolism in foam cells results in inflammatory cytokine production and induces cell death (apoptosis). During the development of atherosclerosis, abundant apoptotic macrophages or neutrophil extracellular traps (NETs) within plaques cannot be removed by surrounding macrophages because of defective efferocytosis, leading to secondary necrosis accompanied by the release of cellular contents such as lipids, cell debris and DAMPs, which ultimately contribute to a lipid-rich necrotic core and unresolved inflammation. Notably, macrophages can be classified into five main subsets based on several scRNA-Seq studies on atherosclerotic plaques in mice; these subsets are namely res-like macrophages, TREM2^hi^ macrophages, inflammatory macrophages, proliferating macrophages and IFNIC macrophages. Among these macrophages, res-like macrophages, with an M2-like phenotype, are distributed in the adventitia. TREM2^hi^ macrophages, with powerful lipid catabolism capabilities, are regarded as foam cells and are likely derived from SMCs. Inflammatory macrophages, with upregulated inflammatory genes and pathway activation, are key contributors to plaque inflammation. Generated by BioRender.

Atherosclerosis, a chronic inflammatory disease involving abnormal lipid metabolism, shares some similarities with autoimmune diseases due to the presence of immune responses directed against self-components within the arterial wall. Autoreactive effector T cells, which recognize self-antigens like oxidized low-density lipoproteins (oxLDL), play a pivotal role in triggering the inflammatory response associated with atherosclerotic lesions. Atherosclerosis involves the formation of lipid-rich plaques in the arterial intima (which also are characterized by a large population of immune, nonimmune, and apoptotic cells and apoptotic cell debris). As the pathological basis of most cardiovascular diseases, e.g., myocardial infarction, stroke and heart failure, with high morbidity and mortality rates, atherosclerosis has emerged as the leading cause of death worldwide [1, 2]. According to the ‘response to injury’ hypotheses, endothelium activation or damage is a crucial step in atherogenesis, involving endothelial dysfunction, increased permeability, formation of focal lesions, and a pro-inflammatory state. This damage can be induced by classical cardiovascular risk factors, including hyperlipemia, hypertension, hyperglycemia, obesity, and arterial wall shear stress [3–6]. Accordingly, the aggregation of low-density lipoproteins (LDLs) in the intima, pathological modifications (oxidation and acetylation), and increased expression of leukocyte adhesion molecules mediate the rolling and firm attachment of monocytes and lymphocytes to the damaged intima surface. After stimulation by chemokines and cytokines, monocytes enter the intima and differentiate into mature macrophages, which are able to ingest excessive lipoproteins and eventually form cholesterol-enriched foam cells, which are characterized by increased expression of lipid-processing genes [7, 8]. Interestingly, although the clearance of lipoproteins by macrophages seems to contribute to the prevention of deleterious lipid production, the excessive lipid metabolism in macrophages leads to alterations in their phenotypes and functions, ultimately causing their death. For example, foam cells cannot easily escape the intima and are thus trapped due to their diminished migration capacity [9, 10], leading to the accumulation and retention of foam cells in the arterial intima. During the progression of atherosclerosis, a large number of foam cells or macrophages undergo apoptosis induced by oxidized LDLs, cholesterol crystals, hypoxia, mitochondrial dysfunction or abundant death ligand protein expression. Apoptotic cells contribute to either immune suppression or inflammation resolution [11, 12]. However, in advanced plaques, efferocytosis is impaired; hence, abundant apoptotic cells are ineffectively eliminated, resulting in secondary necrosis together with the release of their lipid contents, inflammatory cytokines, cellular debris and damage-associated molecular patterns. These released substances not only trigger a stronger immune response to perpetuate plaque inflammation but also constitute the complex plaque microenvironment that contributes to necrotic core formation (Fig. 1).

Considerable attention is being directed to therapeutic strategies for atherosclerosis that are based on macrophage targeting. For example, a phenotypic shift in macrophages (from the M1-like phenotype to the M2-like phenotype) can be achieved by metabolic reprogramming because the metabolic states of macrophages determine their functionalattributes [13, 14]. Notably, M2-like macrophages rely extensively on the mitochondrial oxidative phosphorylation (OXPHOS) system [15], while M1-like macrophages are prone to exhibit anaerobic glycolysis [16].

**Fig. 2:**
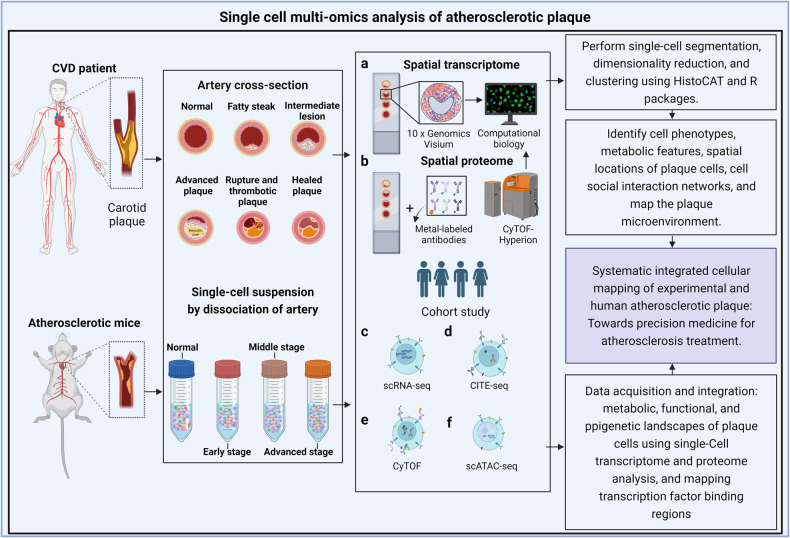
Uncovering the systematic plaque cell formation via experimental and human atherosclerosis studies for the discovery of precision drugs by using single-cell and multiomics approaches. Tissues at different stage of plaque development from atherosclerotic model mice or patients with atherosclerosis-related cardiovascular diseases were processed into tissue sections and single-cell suspensions for single-cell and multiomics analysis. **a**, **b** Using imaging mass cytometry and spactial transcriptomics to map in situ plaque microenvironment information such as cell type, metabolic characteristics and spatial distribution. **c**–**f** Single-cell transcriptome sequencing (scRNA-seq), cytometry by time of flight (CyTOF), cellular indexing of transcriptomes and epitopes by sequencing (CITE-seq) and single-cell assay for transposase-accessible chromatin sequencing (scATAC-seq) were performed to comprehensively map the metabolic, functional and epigenetic landscapes of plaque cells. Generated by BioRender.

Through advanced single-cell multiomics technologies (Fig. 2), scientists are able to distinguish discrete immune or nonimmune cells [17–20] in atherosclerotic plaques and analyse the functions of different plaque cell subsets and their interaction networks. In this review, considering recent advances in understanding atherosclerotic progression in humans and mice, we focus mainly on the origin, heterogeneity, metabolic pathways and death modalities of plaque macrophages in response to the complex plaque microenvironment.

## The natural history of the human atherosclerotic plaques

The natural history of human atherosclerotic plaques in distinct vascular regions reveals a progressive clinical presentation; although some individuals remain asymptomatic throughout their lifespan, others suffer ischemic symptoms or potentially life-threatening events, e.g., acute myocardial infarction or stroke [21]. The current classification of human atherosclerotic plaques includes four different subgroups [3–21]: (i) stable plaques, (ii) vulnerable plaques, (iii) unstable thrombotic plaques and (iv) healed lesions. All stable plaques are characterized by a low level of inflammatory infiltrate [22–25].

• The typical fibrous cap of an atheroma comprises a large lipidic-necrotic core containing extracellular lipids, cholesterol crystals and necrotic cell debris, covered by a thick fibrous cap, whose thickness varies in different vascular regions: <65 µm in coronaries and <165 µm in carotids [26–28]. The amount of inflammatory infiltrate within the fibrous cap is modest.

• Vulnerable plaques, or thin fibrous cap atheromata (TCFAs), are “high risk” plaques, prone to rupture and thrombosis [29]. These plagues are characterized by abundant inflammatory cells, macrophages, T cells and a few smooth muscle cells. Nodules formed by eruptive calcification [30, 31] are large calcified structures protruding into the vascular lumen that form ectopic nodules. The thin fibrous cap frequently ruptures, and an overlying luminal thrombus is formed.

• Unstable plaques are associated with the presence of a luminal thrombus and correlated with the fast progression of atherosclerotic disease and the onset of acute symptoms [28–32]. Acute thrombi might undergo rupture, erosion, or calcification. A disrupted fibrous cap is infiltrated by numerous macrophages and lymphocytes and is associated with an overlying thrombus in continuity with the underlying necrotic core [33, 34]. Similar to other ectopic calcifications [35, 36], two different types of calcifications in carotid atheromatous plaques have been described [37]: hydroxyapatite and calcium oxalate nodules [38]. Among cells in plagues, macrophages are thought to be the main contributors to plaque calcification. Indeed, these cells exhibit strong phenotypic plasticity and differentiate into a variety of cell types depending on the surrounding environment [39]. M1 macrophages can directly release oncostatin M (OSM) to promote the differentiation of vascular smooth muscle cells (VSMCs) with osteoblastic phenotypes through activation of the JAK3-STAT3 pathway, thus promoting the formation of hydroxyapatite structures in plaques [40]. Hemorrhage within a plaque is commonly due to the rupture of small, newly formed and thin-walled vessels, which can easily break following an increase in pressure or the presence of bone spicules or cholesterol clefts. Sometimes, intraplaque bleeding is sudden and large, causing rapid occlusion of the vascular lumen, which leads to acute onset of clinical symptoms.

Inflammation factors and, in particular, different types of monocytes/macrophages play determinant roles in all phases of plaque evolution. M1-like macrophage infiltration has been described in early stages of atherosclerotic lesion development. Moreover, a topographic relationship among the inflammatory infiltrate, plaque rupture and thrombosis play pathogenetic roles in macrophagic cells at cap rupture sites in patients who die because of acute myocardial infarction. In contrast, M2-like macrophages seem to play a prominent role in the organization of thrombi, resulting in transformation into a healed lesion [41].

## Morphological characterization of macrophages

Macrophages play a critical role in the elimination of pathogens, apoptotic cells, and cell debris [42, 43]. Additionally, they actively participate in tissue inflammation and regeneration [44–46]. The alteration of macrophage morphology essentially depends on their activation state or their responses microenvironment stimuli [47, 48]. Specifically, after stimulation with GM-CSF, macrophages differentiated from human monocytes are characterized by a small and round shape morphology. In the presence of M-CSF, macrophages exhibit an elongated shape and contain numerous vacuoles [49]. Understanding the relationship between the morphological characteristics and functional properties of macrophages during the development of diseases will help to effectively predict patient prognosis and further improve therapeutic outcomes [50].

## The origin of macrophages in the plaque

Macrophages are readily recruited to plaque-prone sites and produced through haematopoiesis in the bone marrow [51, 52]. An additional source of plaque macrophages, namely, local proliferating macrophages, contributes to the formation of atherosclerotic plaques [53–55]. Moreover, in the context of atheromatic plaques, intimal vascular smooth muscle cells (VSMCs) have the ability to transdifferentiate into macrophage-like cells. These VSMCs not only take up lipoprotein and become VSMC-derived foam cells but also drive the formation of macrophage-like cells, mesenchymal stromal/stem cells and osteochondrogenic cells by phenotypic switching [56]. Additionally, aortic macrophages originating from yolk sac- and bone marrow-derived monocytes after birth are similar to resident macrophages in different tissues and organs and play crucial roles in maintaining physiological function and tissue homeostasis [57–59]. Thus, the diversity of macrophage sources [60] contributes to the heterogeneity of plaque macrophages.

## The contribution of circulating monocytes to plaque macrophages development

Circulating monocytes in mice are generally classified into two major subsets, conventional Ly6C^high^ monocytes (with high expression of CCR2 and CD62L and moderate expression of CX_3_CR1) and patrolling Ly6C^low^ monocytes (with high expression of CX_3_CR1 as well as low expression of CCR2 and CD62L) [57–59, 61]. The developmental trajectory from haematopoietic stem cells to monocytes or macrophages supports the view that Ly6C^high^ monocytes give rise to Ly6C^low^ monocytes via preferential transition into Ly6C^int^ monocytes named intermediate monocytes [59, 62, 63]. In humans, according to the expression levels of CD14 and CD16, monocyte subsets are classified into CD14^+^ CD16^−^ and CD14^+^ cells, which are thought to correspond to Ly6C^high^, Ly6C^int^ and Ly6C^low^ monocytes in mice, respectively [64, 65].

Interestingly, these monocyte subsets represent heterogeneity not only in the development trajectory but also in physiological function. For example, steady-state Ly6C^low^ monocytes are regarded as endothelial cell-protective monocytes that patrol the arterial vasculature, where they scavenge particles, including lipoproteins, cellular debris, and necrotic cells [66, 67]. An increase in the number of Ly6C^low^ monocytes under hyperlipidaemic and atherosclerotic conditions partially depended on CCR5, and depleting the number of patrolling Ly6C^low^ monocytes via knockout of transcription factor Nr4a1 (Nur77) determined the survival or death of Ly6C^low^ monocytes in ApoE^/−^ mice, resulting in pronounced endothelial apoptosis and exacerbated atherosclerosis, clearly confirming that Ly6C^low^ monocytes are crucial for maintaining endothelial cell homeostasis [67]. Moreover, recent work based on an in-depth phenotype analysis of monocyte subsets demonstrated that LYN (Lck/yes novel tyrosine kinase) regulated the signaling pathways and lifespan in Ly6C^low^ monocytes, and *Lyn* deficiency led to the production of protective patrolling monocytes, impairing atherogenesis induced by a high-fat diet [68].

Conventional Ly6C^high^ monocytes, regarded as the constituents of inflammatory monocyte populations, are able to rapidly respond to inflammatory signals, invade damaged sites, and ultimately differentiate into macrophages [65]. Specifically, in atherosclerosis, steady and sustained recruitment of blood-borne monocytes (Ly6C^high^ monocytes) to lesions accelerates the progression of atherosclerosis, and Ly6C^high^ monocytes are thus thought to be proinflammatory cells [8, 69]. A high level of Ly6C^high^ monocytes in cases of hyperlipidaemia have been associated with monocytosis [69]; these monocytes are preferentially produced in bone marrow and from progenitor cells in the initial stage of atherosclerosis. During atherosclerotic development, the spleen, as an extramedullary site, gradually supplements the haematopoietic function of the bone marrow by generating Ly6C^high^ monocytes that infiltrate plaque regions [51]. Moreover, inflammatory Ly6C^high^ monocytes can be converted into M2-like macrophages in a STAT6-dependent manner, leading to atherosclerosis regression [70].

## The contribution of local proliferation of macrophages to plaque macrophage formation

This is an open question [53, 71, 72]: local macrophage proliferation contributes to ~87% of the macrophages accumulated in advanced atherosclerotic lesions [53]. Recent evidence revealed that aortic intima resident macrophages (Mac^AIR^) arise from bone marrow progenitors and are seeded into the aorta at birth and are sustained mainly through local proliferation in the a steady state, independent of the number of recruited monocytes [71]. Moreover, Mac^AIR^ cells can also become foam cells and promote the recruitment of monocytes, thereby contributing to the formation of early atherosclerotic lesions. Due to their limited proliferation during plaque progression, which is insufficient to drive the expansion of plaque macrophages, Mac^AIR^ cells rely heavily on monocytes for atherosclerotic plaque progression [71]. Interestingly, apoptotic cell degradation after macrophage efferocytosis induces macrophage proliferation, which promotes tissue repair during atherosclerosis regression [73]. Therefore, locally proliferating macrophages play multifaceted roles in different stages of atherosclerosis, and the plaque microenvironment, such as the level of ox-LDL [74], the number of apoptotic cells or their metabolites, may influence their proliferation [73].

## The contribution of smooth muscle cell (SMC) transdifferentiation to plaque macrophages

SMCs have garnered attention for a long time as an important contributor to plaque growth [56]: at least a subset of foam cells in atherosclerosis originates from SMCs [75]. Furthermore, VSMCs coexpress CD68 (as a macrophage marker) and αSMA in human atherosclerotic plaques [76, 77], and SMCs can transdifferentiate into macrophage-like cells after cholesterol loading [78]. SMCs can undergo colony proliferation and be converted into macrophage-like cells that lose αSMA expression and that constitute a primary component of atherosclerotic lesions [55]. However, these data need to be evaluated with caution because macrophage markers (CD68 or MAC2) are also expressed in other myeloid cells, and the possibility of labeled cells differentiating into other cells (mesenchymal stem-like and osteochondrogenic cells) should be considered. Moreover, the transformation of SMCs into macrophage-like cells is governed by diverse factors: deficient Krüppel-like factor-4 (KLF4) in SMCs resulted in reduced SMC-derived macrophage-like cells and a profound decrease in lesion size [79]; BCLAF1 (BCL2 [B-cell lymphoma 2]-associated transcription factor-1) was a central transcription factor for the survival and transdifferentiation of SMCs into a macrophage-like phenotype under lipid stress conditions [80]. In contrast, activation of the NOTCH signaling pathway completely prevented the transformation of SMCs into macrophage-like cells in vitro [81]. Indeed, a portion of plaque macrophages are derived from SMCs. However, the true contribution of SMC transdifferentiation to the macrophage population in atherosclerosis needs to be determined, and the biological functions of these SMC-derived macrophages still need to be further substantiated.

## The landscape and heterogeneity of aortic macrophages in atherosclerosis

Macrophages are a highly heterogeneous and plastic cell population that rapidly respond to microenvironmental signaling. Macrophage subsets were initially described as M1 and M2-like macrophages, which represent two extremes of the activated macrophage spectrum [82–86]. M1-like macrophages are the predominate macrophage cells in the rupture-prone shoulder regions of a plaque, while M2-like macrophages are the most abundant macrophage type in the plaque adventitia [84]. However, macrophages constitute a broad phenotype spectrum and extensive plasticity when exposed to local tissue signaling cues, including signaling by cytokines, pattern recognition receptor ligands, and other immunomodulatory molecules [87, 88].

In addition to M1/M2-like macrophage subsets, Mox, M4, M(Hb) and Mhem macrophages have been described in plaques [85, 89, 90]. Intraplaque Mox macrophages, with decreased phagocytic and chemotactic capacity, are activated by oxidized phospholipids and regulated by a key regulator nuclear erythroid-2 related factor (*Nrf2*), which modulates the expression of redox-regulating genes such as haem oxygenase-1 (*HO-1*), thioredoxin reductase 1 (*TrxR1*), and sulfiredoxin-1 (*Srxn1*) [91]. M4 macrophages, frequently found in the atheromas, are activated by the platelet chemokine CXCL4 [92] and characterized by the coexpression of matrix metalloproteinase-7 (MMP7) and Ca^2+^-binding protein S100A8 [93]. CXCL4-induced M4 macrophages seemingly play a harmful role due to a reduction in the atheroprotective hemoglobin receptor CD163 and a positive association with vulnerable plaques [94, 95]. In contrast, M(Hb) and Mhem macrophages activated by hemoglobin and haeme, respectively, play more atheroprotective roles attributed to their prevention of foam cell formation and lipid accumulation by promoting the expression of reverse cholesterol transport-associated genes, such as liver X receptor alpha (LXRα) and ATP-binding cassette transporters A1 (ABCA1) [96, 97]. Notably, CD163^+^/CD206^+^ (also known as mannose receptor, *Mrc1*) macrophages, M(Hb), often involved in autoimmune response, do not always confer protection against atherosclerosis because CD163^+^ macrophages can promote angiogenesis, vascular permeability and inflammatory cell recruitment via the CD163-HIF1α-VEGFA pathway [98, 99]. These studies emphasized the multifaceted roles of macrophage subsets in determining the plaque inflammatory state and influencing atherosclerosis progression.

## Aortic macrophage subpopulations based on single-cell technologies

**Fig. 3:**
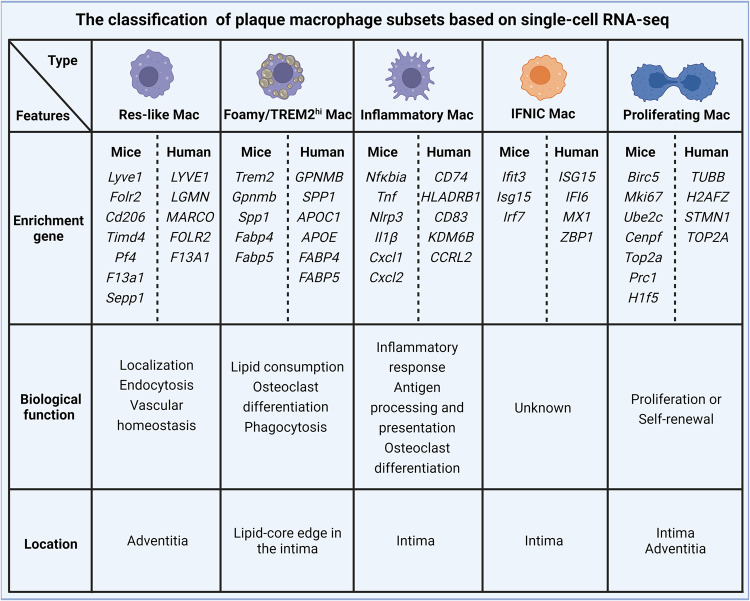
Subpopulation classification of macrophage within plaque based on scRNA-seq. Plaque macrophages can be classified into five main subsets based on several scRNA-seq studies in humans and mice, including Res-like macrophages, Foamy/TREM2hi macrophages, Inflammatory macrophages, Proliferating macrophages, and IFNIC macrophages (Type I IFN induced response signature). These macrophage subsets exhibit distinct gene characteristic, biological functions, and spatial distributions.

There are five main macrophage subsets in the atherosclerotic plaques of humans and mice; these cells include resident-like macrophages (Res-like Macs), inflammatory macrophages (inflammatory Macs), macrophages with high expression of triggered receptor expressed on myeloid cells 2 (TREM2^hi^ Macs), proliferating macrophages (proliferating Macs) and interferon-inducible macrophages (IFNIC Macs) [100] (Figs. 1, 3).

Res-like macs originate from embryonic CX3C motif chemokine receptor 1-positive (*CX3CR1*^+^) precursors and circulating monocytes after birth [101], and these cells are predominantly located in the adventitia of arteries under both healthy and disease conditions [100]. Moreover, single-cell studies revealed that Res-like Mac are involved in the process of endocytosis, and lysosomes and are enriched with proteins encoded by genes such as lymphatic vessel endothelial hyaluronan receptor 1 (*Lyve1*), *Cx3cr1*, *Timd4*, folate receptor β (*Flor2*), *Cd206*, *Ccl8*, factor XIIIa (*F13a1*), carbonyl reductase 2 (*Cbr2*), seleno-protein P (*Sepp1*), colony stimulating factor1 (*Csf1*) and platelet factor (*Pf4*), thereby presenting an M2-like phenotype [7, 101–103].

Inflammatory Macs, regarded as chemokine^high^ macrophages [104] or nonfoamy macrophages [7], not only constitute plaque macrophage populations but are also considered to be the main drivers of inflammation [105–107] in the lesions because of the many inflammatory pathways involved in this subset of cells with high expression of inflammatory genes, including *Ccl2-5*, *Cxcl1*, *Cxcl2*, *Il1β*, *Nlrp3*, *Nfkbia*, *Tnf* and *Ear1* [101], similar to the gene profile of M1-like macrophages. Moreover, higher expression of CCR2 in inflammatory macrophages suggests that these cells are likely derived from circulating monocytes.

TREM2^hi^ Macs are foamy lipid-laden macrophages with high expression of *Trem2*, *Gpnmb*, *Spp1* (secreted phosphoprotein 1), *Mmp12*, *Mmp14, Cd9*, and markers of lipid transport (*Abca1*, *Lipa*, *Fabp4* and *Fabp5*) and lysosomal cathepsins (*Ctsb*, *Ctsd* and *Ctsz*), in atherosclerotic plaques but not in healthy aortas [7, 101, 108]. TREM2^hi^ Macs, constituting a lipid-handling macrophage subset, seem to be endowed with powerful lipid catabolic and anabolic capabilities and present genetic features similar to those of osteoclasts, suggesting a role in plaque calcification. How TREM2^hi^ Macs affect plaque calcification remains unclear. A possible reason is that plastic VSMCs may adapt to the complex plaque microenvironment and thus acquire osteoclastic and macrophagic phenotypes [101, 109]. Notably, although foamy TREM2^hi^ Macs exhibit low expression of inflammatory genes, they do not establish a proinflammatory milieu [7].

Proliferating Macs and IFNIC Macs, two small clusters of macrophages, reside in healthy vessels and atherosclerotic plaques, exhibiting different transcriptional profiles depending on the conditions [7, 71, 103, 104]. These proliferating mac populations are enriched in *Birc5* **(**baculoviral IAP repeat-containing -5, inhibiting apoptosis), Stmn1 (stathmin-1) and Mki67 (marker of proliferation Ki67), reflecting a proliferative state and likely functioning as pools of macrophages to maintain macrophage self-renewal and population numbers. The IFNIC Macs subset displays high expression of the interferon-stimulated genes, including Ifit3, lsg15 and Irf7. It has been reported that IFNIC Macs amplify post-MI (myocardial infarction) inflammation by initiating IRF3 [110]. However, IFNIC Macs are also present in the aorta in a steady state and are regarded as resembling as adventitial macrophages [71]. Therefore, the function of IFNIC Macs in aortic homeostasis or disease progression is still unclear and needs to be determined via further investigation in upcoming studies.

Sanin et al. [111] established a common framework of monocyte-derived macrophage activation, which is also applicable to the analysis of macrophage activation status during atherosclerotic progression and regression. Specifically, distinct macrophage activation stages were defined on the basis of four conserved activation pathways, including the “phagocytic pathway”, “oxidative stress pathway”, “inflammatory pathway”, and “remodeling pathway”, representing P1, P2, P3, and P4, respectively. Moreover, these authors found that phagocytic pathway macrophages are the predominant atherosclerotic plaque macrophages and the most abundant macrophages under a condition characterized by dietary and pharmacological intervention-induced atherosclerosis regression characterized by high expression of phagocytosis- associated genes, suggesting that these interventions accelerated the transition of late P2 stage cells into final P2 stage cells. The biological functions and metabolic changes of macrophages during the course of inflammatory responses and in tissue homeostasis are still under investigation.

## Macrophage interactions with T cells in atherosclerotic plaques

Intercellular interactions within an atherosclerotic plaque orchestrate the pathogenesis of atherosclerosis and associated cardiovascular events [108–112]. Fernandez et al. [108] discovered that adaptive signals and ligands derived from T cells were involved in macrophage activation, polarization, migration, and foam cell formation through ligand‒receptor interactions (VCAN-TLR1/2 and HSP90B1-TLR2, which are involved in the activation of proinflammatory macrophages; APOE-LRP1 and LRPAP1-LDLR, which are involved in regulating lipid, including cholesterol, efflux). Similarly, ligands expressed by macrophages were also predicted to bind CD4^+^ and CD8^+^ T-cell receptors, which may have contributed to T-cell proliferation and the immune response. A ground-breaking study [112] characterized the clonal expansion of plaque effector CD4^+^ T cells that present with an activated cytotoxic phenotype (*GZMA*, *GAMK*) and increased expression of *CD69*, *FOS*, *FOSB* and *JUN*, suggesting antigen-specific activation of CD4^+^ T cells in plaques. Interestingly, the TREM2^hi^ macrophage (foam cell) population was implicated in the clonal expansion of effector CD4^+^ T cells in atherosclerotic lesions mediated via multiple pathways associated with costimulation and immunological synapse formation; the factors involved included CD99, macrophage inhibitory factor (MIF), ANNEXIN, CD6 and CD40. These observations suggest an autoimmune component in atherosclerotic plaques or the circulatory system driven by autoreactive CD4^+^ T cells, apparently following their interaction with foam cells (Fig. 1). Therefore, targeting autoimmune components within plaques and blood and the costimulatory pathways in T cells and macrophages may be promising approaches to the treatment of atherosclerosis-related cardiovascular diseases.

## Microenvironmental cues shape plaque macrophage metabolism and function

**Fig. 4:**
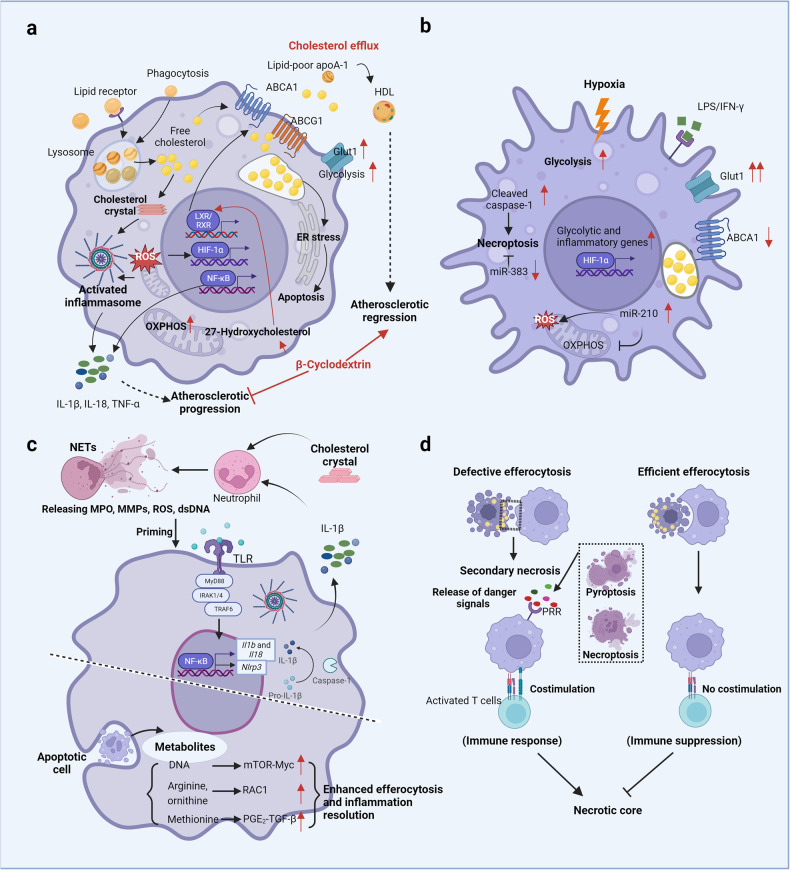
Macrophage metabolism and functions in response to plaque microenvironment development. **a** In atherosclerotic plaques, macrophages are exposed to various lipids (including LDLs, ox-LDLs, oxPAPCs, and cholesterol crystals) that either promote (mostly) or attenuate a proatherogenic microenvironment. Production of intracellular cholesterol crystals and accumulation of free cholesterol following excessive lipid uptake by macrophages via receptor pathways and phagocytosis directly or indirectly lead to metabolic changes (i.e., increased glycolysis and OXPHOS rates), the production of proinflammatory cytokines and the activation of death pathways, ultimately resulting in atherosclerotic progression, which is blunted by effective cholesterol efflux. For example, β-cyclodextrin promotes atherosclerosis regression by activating LXR-targeting genes (such as ABCA1 and ABCG1) that mediate cholesterol metabolism and inflammatory responses in macrophages. **b** Moreover, hypoxic microenvironments jointly created by increased oxygen demand in inflammatory cells and vigorous metabolism (such as that in activated macrophages and lipid-loaded foam cells) inversely potentiate glycolysis, inflammatory gene expression, and necroptotic death in macrophages. **c** Cholesterol crystals can also trigger NETosis and the release of danger signals (MPO, MMPs, ROS, dsDNA) that prime macrophages for cytokine release (IL-1β). Recent works have shown that metabolites (including DNA, arginine, ornithine, and methionine) released from apoptotic cells during efferocytosis enhance continuous efferocytosis and injury resolution through different signaling pathways. **d** Additionally, macrophages in the plaque undergo distinct death modes (i.e., apoptosis, necroptosis, and pyroptosis), which influence atherosclerotic progression and plaque stability. The elimination of apoptotic cells via efficient efferocytosis induces immune suppression, while defective efferocytosis induces immune responses triggered by danger signals from necroptotic or pyroptotic cells. Generated by BioRender.

Here, we outline the microenvironmental factors driving alterations in macrophage functions alongside metabolic adaptation based on in vivo evidence and in vitro mechanistic research (Fig. 4).

## Lipid-mediated changes in macrophage metabolism and functions

Monocyte-derived and tissue-resident macrophages in the intima take up lipoproteins that are then retained and modified via diverse processes, including efferocytosis, micropinocytosis, and phagocytosis as well as through scavenger receptors (CD36, SR-A, SR-B1, LRP1, and LOX1) [8]; these modifications are beneficial changes that eliminate inflammatory lipids. However, excessive uptake of lipids by macrophages contributes to their transformation into foamy macrophages, leading to inhibited migration and entrapment in the intima, where free cholesterol-induced apoptosis of macrophages further results in the amplification of chronic inflammation [8].

An in vitro study mimicking macrophage foam cell formation via ox-LDLs showed that ox-LDLs increased the uptake of [16] F-fluorodeoxyglucose by macrophages and enhanced glycolysis in macrophages by upregulating GLUT1 and hexokinase expression. Mechanistically, this metabolic alteration was mediated by Nox2-dependent production of reactive oxygen species (ROS), which promote hypoxia-induced factor-1α activation and stabilization [113]. Similarly, Chen et al. [114] found that ox-LDL/CD36 signaling contributes to a metabolic shift from the mitochondrial oxidative phosphorylation (OXPHOS) to glycolysis, superoxide production, and proinflammatory cytokine expression in peritoneal macrophages ex vivo. In addition, increased rates of glycolysis and macrophage foam cell formation and inflammation induced by ox-LDLs were associated with pyruvate kinase M2 (PMK2), suggesting a close link between inflammation-related cellular dysfunction and metabolic changes in the context of atherosclerotic coronary artery disease [115]. Specifically, PKM2 induced by ox-LDLs interacts with sterol regulatory element-binding proteins (SREBP1) and increases lipid binding and uptake or inhibits cholesterol efflux, ultimately leading to the formation of foamy macrophages. Consistent with the aforementioned study, upregulated PKM2 expression was evident in atherosclerotic plaques in Ldlr^−/−^ mice fed a high-fat Western diet compared with control mice fed a chow diet, and lack of PKM2 in myeloid cells reduced atherosclerotic lesion progression attributed to inhibited inflammation, impaired glycolytic activity, or enhanced efferocytosis that is typically mediated via the upregulation of LRP1 in macrophages [116].

Internalized lipoproteins and their associated lipids are digested in lysosomes, which subsequently release a large amount of fatty acid and free cholesterol. In addition, free cholesterol can be pumped out from cells to APOA1-deficient cells, with low lipid synthesis via ABCA1 and ABCG1, key lipid transporters regulated by the transcription factor heterodimeric-liver X receptor (LXR)–retinoid X receptor (RXR). Activation of this reverse cholesterol transport pathway leads to the accumulation of cytotoxic free cholesterol, which can traffic to the endoplasmic reticulum (ER) and activate the unfolded protein response (UPR), eventually resulting in ER stress-CHOP-Bcl2-associated X protein-mediated apoptosis in macrophages [117, 118]. Furthermore, enrichment of free cholesterol in foamy macrophage membranes can boost inflammatory signaling from lipid rafts, particularly via the activation of the TLR4 and NF-κB signaling pathways [119, 120]. Finally, cholesterol crystals formed by free cholesterol in macrophages can trigger NLRP3 (NOD-, LRR- and pyrin domain-containing 3) inflammasome activation, which results in the secretion of IL-1β and IL-18. Accordingly, a Western diet or high-fat diet can induce “innate immunity reprogramming” in an NLRP3-dependent manner [121, 122]. In this scenario, ox-LDLs can induce trained immunity in human monocytes via epigenetic histone modifications; these cells are characterized by increased proinflammatory cytokine expression and enhanced formation into foam cells [123]. Interestingly, neutrophil extracellular trap formation triggered by cholesterol crystals licenses macrophages for the production of cytokines, which activate T helper 17 cells and lead to immune cell recruitment and amplification of immune responses in plaques [124, 125].

In addition to ox-LDLs, oxidized phospholipids composed of polyunsaturated fatty acids, which are also recognized and taken in by receptors (including CD36, SR-B1, Toll-like receptor (TLR)-2), TLR4, and E-type prostaglandin receptor (EP2)) and nonreceptors [126, 127], exhibited proinflammatory functions and atherogenicity in hypercholesterolaemic mice [128, 129]. In BMDMs primed with LPS, oxidized phospholipid 1-palmitoyl-2-arachidonyl-sn-glycero-3-phosphorylcholines (collectively known as oxPAPCs) potently drove a hypermetabolic state characterized by increased glycolysis and OXPHOS as well as glutaminolysis and oxaloacetate accumulation. These metabolic changes potentiate HIF-1α stabilization and further promote hyperinflammation by increasing IL-1β production [130]. Furthermore, oxPAPC-driven immunometabolic adaptation also occurs in atherosclerotic mice and hypercholesterolemic patients [130].

These findings support the notion that lipid-loaded foamy macrophages are proinflammatory cells that drive atherosclerotic lesion progression. However, lipid accumulation in macrophages does not always lead to activation or inhibition of inflammatory gene expression. For example, foamy macrophages in which desmosterol, a cholesterol biosynthetic intermediate, had accumulated, induced many homeostatic responses, including activation of LXRα/β gene expression, inhibition of SREBP1 and SERBP2 gene expression, alteration of fatty acid metabolism [131–134], and suppression of inflammatory response-related gene expression [135]. Subsequent research showed that depletion of desmosterol in macrophages promoted IFN-mediated responses and attenuated the expression of anti-inflammatory macrophage markers. Moreover, increased mitochondrial ROS production and NLRP3 inflammasome activation were observed in the desmosterol-depleted macrophages [136]. These clear discrepancies may be well explained by the results of recent single-cell studies on atherosclerosis, which revealed that nonfoamy but not foamy macrophages exhibited proinflammatory effects with distinct features that are also found in macrophages in atherosclerotic plaques [7]. Intimal foamy macrophages resemble TREM2^hi^ macs by exhibiting high expression of genes involved in cholesterol metabolism, fatty acid transport, OXPHOS, proteasome and lysosome activity and PPAR signaling.

## Hypoxia-driven changes in macrophage metabolism and functions

Hypoxia is evident in all stages of atherosclerotic lesion formation [137–140]. An imbalance between the supply and demand of oxygen [141–143] in the arterial wall leads to a hypoxic plaque microenvironment, mainly arising from the combination of increased cellular metabolic demand and reduced oxygen diffusion caused by thickening of the arterial wall during the development of atherosclerotic lesions [144]. Hypoxia is more likely to be caused by a combination of increased metabolic oxygen demand, especially in metabolically active inflammatory cells, not by impaired oxygen delivery because hypoxia is also evident in some subluminal (20–30 µm) foam cells or areas [138, 139], despite their location of these cells or areas being within the regions with oxygen diffusion (at distances of 100–250 µm [145]).

In humans, hypoxic regions colocalize with HIF, VEGF and macrophages and foam cells, and this colocalization is correlated with intraplaque angiogenesis [138]. In parallel, the expression of HIF-1α target genes (e.g., GLUT1, GLUT3 and HK1) is significantly increased during the progression from early to advanced atherosclerosis development, reflecting enhanced reliance on glycolysis in macrophages [138]. In mice, hypoxic areas contained abundant HIF-1α-expressing macrophage foam cells with high expression of GLUT1 [139]. In contrast, HIF-1α deficiency in myeloid cells not only led to reduced plaque and necrotic core sizes [146] but also inhibited GLUT1 expression in foam cell-rich plaque regions, which blunted hypoxia-mediated glucose uptake by macrophages in vitro. In addition, hypoxia induced the accumulation of sterol and decreased cholesterol efflux via ABCA1 in macrophages, which was substantially reversed by blocking HIF-1α expression [147]. Apparently, this metabolic switching [148] and proinflammatory activation in macrophages is attributable to the transcriptional activity of HIF-1α, which induces the expression of glycolytic enzymes and glucose transport receptors and simultaneously activates the production of inflammatory proteins. Interestingly, a shift in glucose metabolism towards glycolytic flux in response to hypoxia relies on appropriate mitochondrial ROS levels because ROS generated via mitochondrial complex III are essential for the stabilization of HIF-1α [149]. Similarly, ROS-mediated HIF-1α activation has been found in ox-LDL-stimulated macrophages [113], indicating that HIF-1α activation is jointly regulated by multiple factors such as lipids and hypoxia in plaques. Hence, hypoxia potentiates IL-1β and NLRP3 expression in LPS-stimulated human macrophages [150], and elevated IL-1β production mediated by HIF-1α is evident in macrophage-rich hypoxic plaque regions displaying increased HK2 and cleaved caspase-1 levels [150]. HIF-1α deficiency in macrophages results in the acquisition of certain phenotypes, including low inflammation-related gene [151–153] (e.g., *Mcp1*, *osteopontin* and *iNOS*) expression in vitro [139, 140, 144–146] and reduced macrophage necroptosis rates by regulating miR-210 activity (inhibiting oxidative phosphorylation and enhancing mitochondrial ROS production) and miR-383 activity (increasing ATP levels and inhibiting necroptosis) in vivo [146]. Collectively, these findings highlight that HIF-1α is a key metabolic regulator that accelerates atherosclerotic progression (Fig. 4b).

## Macrophage death versus immune suppression in the plaques

The death of macrophages, cells thus considered “the walking dead” [154], is induced via distinct modalities, e.g., apoptosis, pyroptosis, necroptosis [154, 155], for details see [156]. In the atherosclerotic context, distinct factors contribute to apoptosis, e.g., TNFα, NO, hypoxia, cholesterol crystals, and ox-LDLs [157]. Depletion of p53, which regulates cell death [158–160], in macrophages resulted in deteriorative atherosclerosis in APOE3-Leiden transgenic mice [161]. Under homeostatic conditions, these apoptotic macrophages were efficiently cleared by surrounding macrophages through efferocytosis, leading to immune suppression or inflammation resolution [162]. Notably, apoptotic cell-derived metabolites and products (such as methionine [163], arginine [164], and DNA [73]) play vital roles in the efferocytosis-mediated resolution of inflammation [165] (Fig. 4c). For example, nucleotides produced by the hydrolysis of apoptotic cell DNA via phagolysosomal DNase2a activated the DNA-PK-mTORC2-Myc proliferation-related pathway, which expanded the pool of inflammation-resolving macrophages in vitro and in mice [73]. Moreover, apoptotic cell-derived arginine and ornithine are converted into putrescine, which promotes continual efferocytosis and inflammation resolution by enhancing Rac1 activation [164]. In advanced atherosclerotic contexts, the combination of accumulating apoptotic cells, lipid metabolism dysfunction, and defective efferocytosis in plaques results in secondary necrosis of uncleared apoptotic cells and the release of a large number of cellular components (i.e., DAMPs) into the plaque milieu, which contributes to lipid-rich necrotic core formation and vulnerable plaques. Cell death, a major outcome of advanced atherosclerotic plaques, is mediated by different cell death modalities [166–171], including necroptosis, pyroptosis, and ferroptosis (for an excellent recent review, see [155]) (Fig. 4d).

RIPK3 and MLKL have been demonstrated to mediate macrophage necroptosis during atherosclerosis development. In one study, macrophage-reduced primary necrosis and necrotic areas were discovered in advanced lesions in RIP3^−/−^ LDLR^−/−^ or Apoe^−/−^ mice [170], as well as in MLKL-deficient Apoe^−/−^ mice [171]. Cumulatively, the data suggest that RIPK1 is emerging as a master switch that controls inflammation and cell survival in atherosclerosis progression, and phosphorylation of RIPK1, RIPK3 and MLKL are definitive indicators of necroptosis activation. Pharmacological inhibition of the necroptosis pathway (e.g., necrostatin-1) may be an exciting therapeutic approach to atherosclerosis [172, 173].

Pyroptosis is also involved in atherosclerosis [174, 175]: ox-LDLs, cholesterol crystals, hypoxia-response factors, ROS, DAMPs, and PAMPs promote activation of the NLRP3 inflammasome, which likely drives the pyroptosis of macrophages and induce plaque instability [176–178]. Caspase-11 accelerates atherogenesis and ox-LDL-induced macrophage pyroptosis, which is reversed when the activation of GSDMD is blocked through caspase-11 deficiency [176]. In addition, the absence of melanoma 2 (AIM2) inflammasome-mediated pyroptosis and inflammation in macrophages have been demonstrated to exacerbate atherosclerosis in clonal hemtopoiesis [179]. Interestingly, this AIM2-dependent form of inflammation-aggravated atherosclerosis, with accelerated necrotic core formations was reversed by knockout of caspase-1/11 or GSDMD [179]. Therefore, targeting inflammasomes or the pyroptosis executioner GSDMD can inhibit atherosclerotic progression and facilitate plaque regression.

## Conclusion

The metabolic characteristics of macrophages and their polarization not only influence the inflammatory responses within a lesion but also play a pivotal role in determining atherosclerosis progression or regression, thus providing valuable insights into the dynamic nature of atherosclerosis [180, 181]. In principle, the modulation of macrophage function can be exploited for the intervention of disease by reducing plaque progression and stabilizing vulnerable lesions. Accordingly, both efferocytosis and β-cyclodextrin have been investigated [182, 183]. Although at very high pharmaceutical doses, β-cyclodextrin treatment reduced plaque size and facilitated atherosclerosis regression through macrophage reprograming in ApoE^−/−^ mice [184]. β-cyclodextrin promoted the consumption of free cholesterol to generate 27-hydroxycholesterol, which in turn activated LXR-targeted gene expression, contributing to enhanced cholesterol efflux and acquisition of anti-inflammatory properties in macrophages (Fig. 4a). The identification of novel therapeutic targets to regulate the metabolism and polarization of macrophages at plaque sites (Fig. 2) may lead to innovative therapies for cardiovascular disease in addition to the traditional management of risk factors and the use of standard-of-care lipid-lowering drugs, leading to effective precision medicine [185].

## Availability of data and materials

Not applicable.
